# Conformational Self-Poisoning in Crystal Growth

**DOI:** 10.1021/jacsau.5c00043

**Published:** 2025-03-18

**Authors:** Yumin Liu, Veselina Marinova, Roger J. Davey, Benjamin Gabriele, Matteo Salvalaglio, Aurora J. Cruz-Cabeza

**Affiliations:** a Department of Chemical Engineering, University of Manchester, Manchester M13 9PL, United Kingdom; b Beijing National Laboratory for Molecular Sciences, Key Laboratory of Organic Solids, Institute of Chemistry, Chinese Academy of Sciences, Beijing 100190, China; c Thomas Young Centre and Department of Chemical Engineering, University College London, London WC1E 7JE, United Kingdom; d Department of Chemistry, Durham University, Durham DH1 3LE, United Kingdom

**Keywords:** crystal growth, molecular flexibility, self-poisoning, growth inhibition, metadynamics

## Abstract

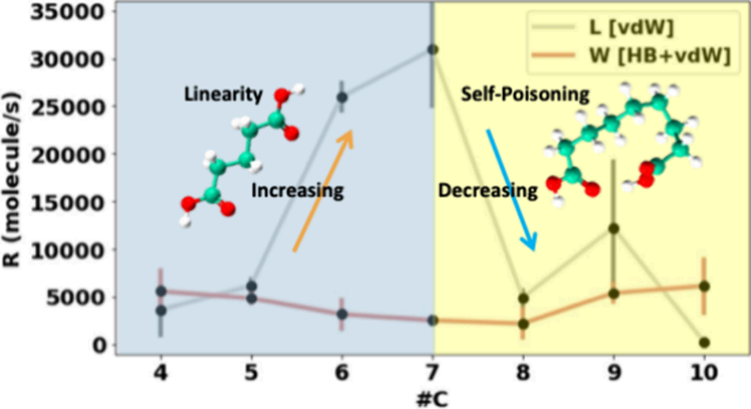

With the ever-increasing complexity of new drug compounds, their crystallization is becoming more challenging than ever. Controlling the crystallization of present and future drugs will remain a chimera unless we gain an improved understanding of the effects of molecular flexibility on crystal nucleation and growth at the molecular level. As a contribution to this understanding, we report here the growth kinetics of a series of diacids with chain lengths from 4 to 10 carbon atoms. These compounds are ideal for such a study since (a) they all crystallize as linear conformers, (b) their crystal structures are very similar across the series, and (c) their molecular flexibility increases with increasing chain length. Upon analysis of their crystal growth behavior, we stumbled upon a surprising finding: the growth of these crystals along the length increases linearly for the series up to the diacid containing seven carbon atoms, beyond which the rates drop dramatically. Such a dramatic decrease in growth rates at longer chain lengths cannot be explained by the crystal structure differences of the diacids. To gain further insights, we explored the conformational landscapes of two diacids in solution using well-tempered metadynamics simulations. With increasing chain length, the conformational landscape becomes more complex, with folded conformations becoming more important for long chain acids. Our simulations show that some of the minor conformers present in the solution act as potent crystal growth inhibitors (a phenomenon we refer to as conformational self-poisoning). To the best of our knowledge, this work represents the first report of conformational self-poisoning in crystal growth, with experimental evidence supported by a molecular-level mechanism. While this effect is bad news for crystallization scientists, who must work with complex flexible compounds, for these diacids, we show that selected solvents are able to disfavor the problematic conformers in the solution, turning off the self-poisoning effect.

## 1 Introduction

Crystallization, the arrangement of approximately 10^23^ molecules into a crystal, is a complex molecular process involving the formation of critical crystal nuclei and their subsequent growth. Many compounds can form crystals, from simple molecules like water to complex proteins, with many consumer products containing crystals of compounds of intermediate complexity.^1^ While the fundamental principles and theories of crystallization are common across all systems, the complexity of the process can increase with the complexity of the molecules. This is especially true for those compounds able to isomerize in solution via tautomerization,^2^ charge transfer,^3,4^ or simple bond rotation.^5^ Different isomers can lead to different crystal forms, some of which offer unique challenges in crystallization.

In the case of tautomerism, the example of barbituric acid is revealing. Until 2011, all known solid forms of barbituric acid contained only its stable triketo tautomer. A solvent-free milling experiment, however, later led to its most stable polymorph, which contains the enol tautomer.^6,7^ The enol tautomer is significantly higher in energy than the triketo one, making the stable polymorph much more difficult to obtain from solution.^6,7^ While most compounds do not have the necessary chemistry to undergo isomerization through a tautomerization reaction or form zwitterionic isomers,^2^ the majority of compounds exist as multiple conformers. Biologically active compounds typically have between five to seven rotatable bonds, with the flexibility of new drugs consistently increasing over the years.^1^ With each rotatable bond leading to two to three distinct conformers in solutions, most midsized molecules are able to populate thousands of different conformers in the crystallizing environment, only one (or a few) of which is able to assemble into crystals. A consequence of this is that molecular flexibility can lead to conformational polymorphism^8^ as well as a decreased tendency for crystallization,^5,9,10^ with some flexible compounds only existing as amorphous solids.^11^

The link between molecular flexibility and crystallization was first made by Yu et al.^5^ in 2000, who introduced the term “crystallization tendency” when referring to compounds. Others followed with the terms “crystallization propensity” and “crystallizability” as a way of qualitatively assessing how “easy” or “difficult” is the process of making a crystal of a given compound.^9,10^ In all those studies, flexible compounds were identified as harder to crystallize than rigid compounds. Attempts followed to try and quantify “crystallization tendency”. Baird et al. studied the melt crystallization ability of 51 molecules and developed a classification system concluding that low-molecular-weight and low-flexibility compounds are generally easier to crystallize.^12^ Threlfall et al. used metastable zone widths and induction time measurements as a way to quantify “crystallization ability”.^13^ We have used both nucleation rates and growth rates as an effective way of quantitatively comparing the kinetics of crystallization of rigid and flexible compounds.^14,15^ In doing so, we have had to carefully question the correct way of comparing kinetics across different systems and have found that to be able to link the observed kinetics to meaningful processes at the molecular level, the rates need to be normalized to account for differences in solubilities and crystal packing.^14,15^ Even comparing the kinetics of growth across polymorphs of the same compound can be a complex exercise.^16^

**Figure 1:**
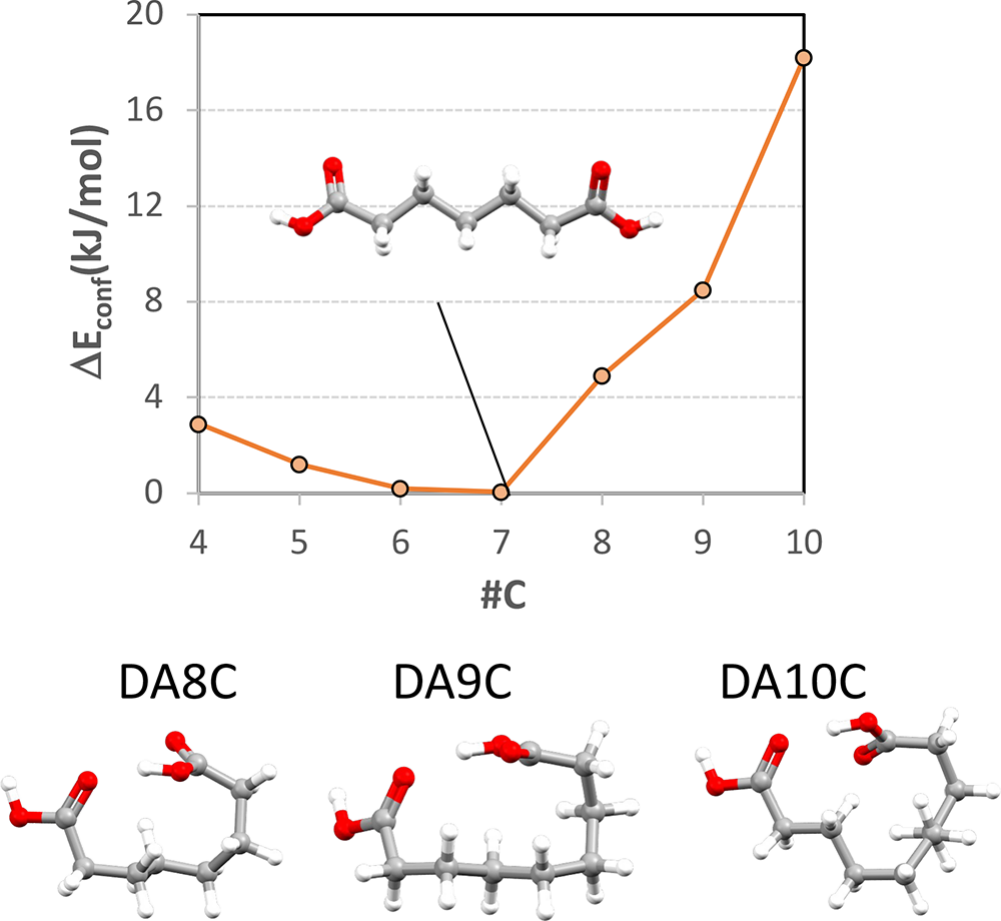
Conformational energy of the linear crystal conformer relative to the lowest energy conformer (Δ*E*_conf_) in ethanol calculated at the M-GGA TPSS/TNP level of theory (COSMO model). The lowest energy conformers for DA7C to DA10C in ethanol are shown in the graph as an illustration.

Continuing our quest for understanding the impact of molecular flexibility on crystallization, we have chosen the series of α,ω-dicarboxylic acids (DA#C) having carbon atoms (#C) from 4 to 10. These are succinic, glutaric, adipic, pimelic, suberic, azelaic, and sebacic acid (DA4C to DA10C). Our rationale for this was simple—while the crystal structures of all the molecules in this series are built from linear chain conformers, with increasing chain length, we imagined that more stable cyclized H-bonded conformers might be possible and dominate the solutions, thus perhaps ultimately impacting crystallization abilities in these series. To justify further work on this system, we first explored the static conformational landscape of the diacids in ethanol using a conformer generator and DFT calculations (Figure 1). As the chain length in our diacid series increases, so does the flexibility of the compounds from 5 rotatable bonds in DA4C to 11 rotatable bonds in DA10C. While there is a strong preference for these diacids and other alkanes to adopt linear conformations in the crystalline state (with rare exceptions^17^), the number of conformations plausible under the conditions of the simulations (implicit ethanol solvent) increases exponentially as the chain length increases. Figure 1 shows the energy of the linear conformer (required for incorporation into the crystal and thus for crystal growth) relative to the global minimum conformer as a function of the DA number of carbon atoms, as calculated with DFT using an implicit ethanol COSMO model. The geometry of the global minimum conformer for each of the DAs with seven carbon atoms and above is illustrated. From DA8C and above, the most stable conformers in alcohol solution are all nonlinear, being stabilized by the formation of an intramolecular H-bond. These cyclized stable conformers are not possible for DA7C and below because the alkane chain is not long enough for the intramolecular acid···acid hydrogen bond to form. Below seven carbon atoms, the linear conformers are all either the most stable or within 4 kJ/mol from the global minimum (or RT), while above seven carbon atoms, the linear conformers become significantly higher in conformer energies. This suggests that as the chain length increases, the population of the “right” conformers for crystallization, the linear conformers, decreases significantly.

This preliminary study thus justified further work on this system, and experiments were then performed in which we grew single crystals of each of the diacids from DA4C to DA10C and measured their growth rates in isopropanol (IPA) and other organic solvents. Finally, to support these experimental results, we expanded on the insights from Figure 1 by carrying out sophisticated modeling to explore the dynamic conformational landscape of these molecules in IPA. The results and conclusions of these studies are reported below.

## 2 Results

### 2.1 Diacids and Their Crystal Structures

Here we study the growth of the β polymorphs of α,ω-alkane dicarboxylic acids with total carbon numbers between 4 and 10. The Cambridge Structural Database (CSD)^18^ refcodes matching the experimental forms used in this work are SUCACB11, GLURAC04, ADIPAC13, PIMELA06, SUBRAC05, AZELAC05, and SEBAAC07, respectively. This is an attractive series to choose since growth is characterized by the formation of hydrogen bonds and chain–chain stacking in orthogonal directions. All these diacids crystallize with *Z*’ = 0.5, with the odd ones crystallizing in the *C*2/c and the even ones in *P*2_1_/c symmetry groups (the stable beta forms). For the odd diacids, the molecule sits on a screw axis (central carbon atom), and the infinite hydrogen bond (HB) chains are constructed through crystallographic inversion and translation. For the even acids, the molecule sits on an inversion center (central bond), enabling periodic HB chains to be constructed through pure translation (Figure S1). For both odd and even acids, chain–chain stacking occurs through translation symmetry along the shortest crystallographic axis (the *b*-axis). The acids have been reported to show odd–even effects across a number of properties, including heat of melting,^19^ solubility,^20^ and mechanical properties.^21^

### 2.2 Single Crystals, Morphologies, and Growth Directions

**Figure 2:**
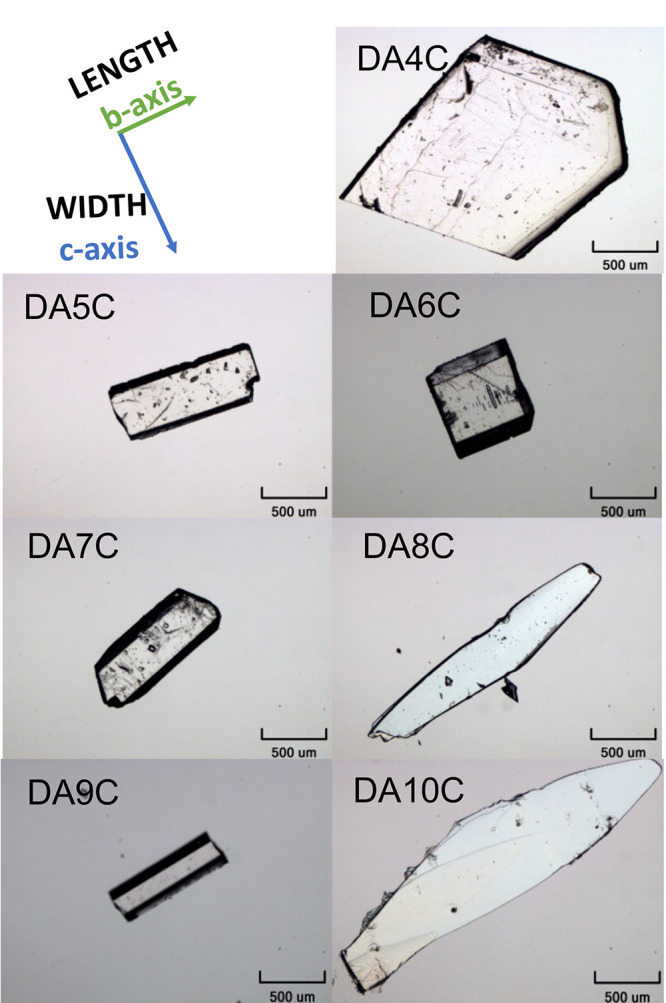
Micrographs of the seed single crystals used for growth kinetic measurements. The (100) faces are dominant faces for all morphologies. Growth rates were measured along the *b* and *c* axis, corresponding to the length and width dimensions of the crystals, respectively.

The growth of good quality single crystals of all diacids was not an easy task especially since the solubility of the larger ones is very low, and they tend to form thin plates that stack and agglomerate along the shortest axis. Consequently, a range of solvents had to be explored to produce good quality crystal seeds by slow evaporation (see the Methods section). Grown seeds of the odd diacids were elongated blocks, while seeds for the even ones were more platelike, with DA8C and DA10C being thin plates (Figure 2). Overall, an increase in chain length leads to an elongation of the crystal length in both odd and even acids. In all cases, face indexing showed that the dominant slowest-growing face (corresponding to the crystal thickness) is aligned with the *a*-axis, with its (100) face being dominant. The width and length dimensions of all systems align with the *c* axis and the *b* axis, respectively (Figure 2). The face indexing agrees with previous morphological studies of adipic^22^ and succinic acids,^23^ and the interactions involved in these three growth directions are further analyzed below.

### 2.3 Underlying Intermolecular Interactions

**Figure 3:**
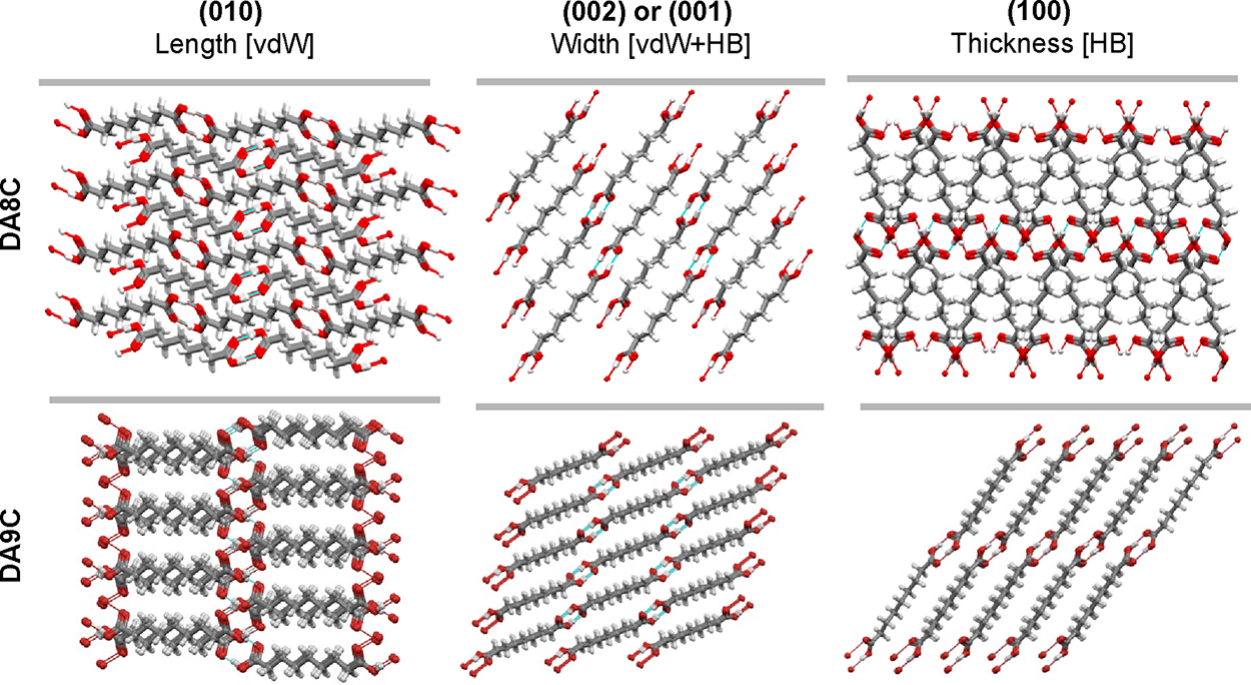
Packing of the (010), (002), and (100) surfaces (corresponding to the length, width, and thickness, respectively) of suberic acid (DA8C) and azelic acid (DA9C). Crystal structures with CSD refcodes SUBRAC05 (DA8C) and AZELAC07 (DA9C).

To link crystal morphologies with underlying structures, we explored the packing and surface properties of dominant crystal surfaces expressed at the length-(010), width-(001)/(002), and thickness-(100) of the diacid crystals (Figure 3). The angle between the direction of propagation of the H-bonded (HB) chains and a vector normal to the facet of interest was calculated (α_(hkl)-[HB]_, values in the Supporting Information (SI)). This angle was found to be 0° along the crystal length for all systems, α_[010]-[HB]_ = 0°, illustrating that the HB chain interactions make no contribution to the layer-to-layer length growth. Visual inspection of the packing of the (010) face also shows this clearly (Figure 3 left), with van der Waals (vdW) interactions dominating the layer-to-layer attachment while HB chains dominate the intralayer interactions. For the width and thickness, the situation is mixed, with α_(hkl)-[HB]_ angles calculated to be, on average, around 45°, with specific angles being system-dependent (see the SI). The (002)/(001) faces (Figure 3, center), important for the crystal width, are terminated by the carboxylic acid groups tilted relative to the surface with angles between 0 and 70°. The layer-to-layer width growth thus involves a mixed contribution from HB and vdW interactions. The (100) face (Figure 3 right), important for the crystal thickness, corresponds to the major (and hence the slowest growing) surface of the crystal morphologies. This surface is highly polar since it is dominated by the carboxylic acid groups. As such, growth along this direction [100] requires molecules to mostly attach through a carboxylic acid dimer formation.

**Figure 4:**
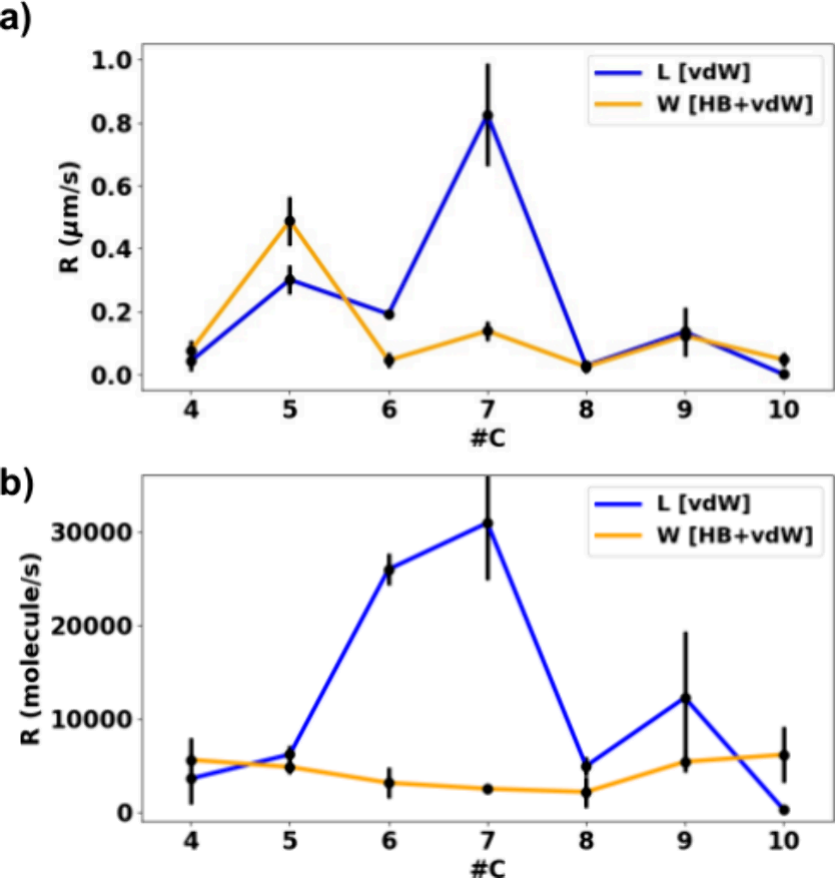
(a) Linear growth rates in μm/s (a) and (b) in molecule/s for all diacids in IPA at 20 °C.

To summarize and simplify in aid of the molecular-level interpretation of the growth data, it is clear that the layer-to-layer growth along the length dimension (*L*) of the diacid crystals is vdW dominated, the width (*W*) is mixed (HB + vdW), and the thickness (T*)* is HB dominated (Figure 4). For the intralayer growth, the opposite interactions dominate in each of the crystal dimensions.

### 2.4 Growth Rates of Crystal Length and Width Dimensions

Prior to the growth rate measurements, solubilities for all systems were measured at 20 °C in IPA and compared with those in various other solvents reported in the literature (see the SI).^20^ As previously observed, the solubilities display odd–even effects. Growth rates along the *L* and *W* dimensions were then measured for all systems in IPA at 20 °C and a supersaturation ratio (*S*) of 1.25. Figure 4a shows the linear growth rates (μm/s) for all diacids along the crystal *L* and *W*. The linear growth rate trend aligns with the solubilities, also reflecting odd–even effects. Additionally, it is evident that odd carbon-numbered diacids tend to grow faster than their neighboring even ones, which is also in line with the solubilities, with a drop in the trend from the data at DA8C onward.

To derive meaningful molecular-level comparisons of rates across different systems (as discussed in a previous work,^14^ also in the SI), linear rates must be first normalized by the solubility (in mole fraction) and then by the step height (*d*_(hkl)_) normal to the growth direction. This acknowledges that for different molecules and structures, the solubility and the step height will change and that this inevitably impacts the rates directly. In this way, we can derive normalized growth rates, which have units of molecule/s (Figure 4b).

Upon such normalization of the linear growth rates (in μm/s), the data paint a different picture. First, we notice that the odd–even effect essentially disappears for W growth, becoming independent of diacid chain length (Figure 4b). Second, the growth of molecules along *L* is mostly greater than that along *W* (with the exceptions of DA4C and DA10C). Third, growth along the *L* direction is intriguing: initially, the *L* growth rate increases significantly from DA4C to DA7C, but beyond this chain length, the rates decrease dramatically. This is a surprising effect since the *b*-axis layer-to-layer growth (Figure 4) is dominated by the stacking of the linear chains, and thus, one expects this to increase as the chain length increases. Therefore, the sharp decrease in *L* rates at DA8C is an interesting observation worth further investigation.

### 2.5 Attachment Energies and Expected Growth Rates

**Figure 5:**
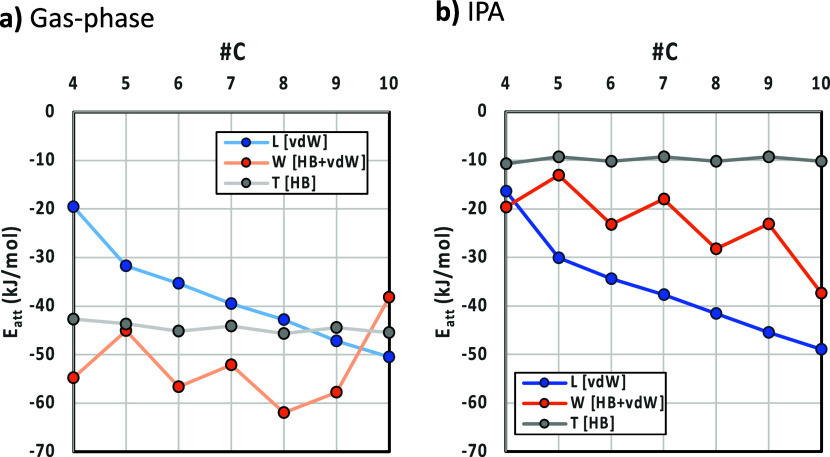
Gas-phase (a) and IPA-corrected (b) attachment energies (*E*_att_) for the diacid series along the *L*, *W*, and *T* of their crystal morphologies.

As an aid to rationalizing the experimental observations of growth rates, it was considered that, in line with classical thinking, the relative growth rates in the different directions might be estimated based on calculated attachment energies. The attachment energy is proportional to the energy released per mole of diacids as one layer of molecules is grown on to a given (hkl) surface. It quantifies the layer-to-layer interactions (HB + vdW) responsible for crystal growth. Figure 5 shows the computed attachment energies (*E*_att_) in the gas phase and in IPA along the main crystal dimensions of the diacids. For these simulations, the (010), (001), and (100) faces were modeled for the *L*, *W*, and *T*, respectively.

First, we focus on the gas phase versus IPA attachment energies. In the gas phase, *E*_att_ is more negative for the *W* direction followed by *T* and then *L*. This trend does not reproduce the expected link between attachment energies, rates, and resulting crystal dimensions (where the *L* growth should be fastest and thus have the lowest *E*_att_)_._ Correction of the *E*_att_ for the solvent (IPA in this case) leads to the correct ranking of attachment energies and rates, with the *L* dimension showing the lowest *E*_att_ (fastest growth) followed by *W* and finally the thickness *T*. As expected, the faces with higher contributions of polar interactions (present in the HB dimers, *W*, and especially *T*) are significantly reranked upon correction of energies for the solvent environment.

The face containing the major contribution from HB layer-to-layer interaction (*T* dimension corresponding to the (100) face) shows a flat trend for the diacid series. This is expected since the layer-to-layer growth here must indeed be controlled by the acid–acid HB dimer, and this interaction remains constant across the entire diacid series. The face containing the major contribution from vdW stacking of chains, the *L* dimension with the (010) face, by contrast, shows a steady, continuous decline in *E*_att_ as the chain length increases in the series. This is also to be expected since as the chain of the length grows, the vdW interaction per molecule should also become more stabilizing, and thus, the rate of growth involving the chain stacks along the *L* dimension of the crystal should increase. The experimental trend follows this up to DA7C. However, beyond this chain length, the sharp drop observed at DA8C breaks the trend. This cannot be explained on the basis of the attachment energies and hence needs further investigation. Finally, the face containing mixed contributions of HB and vdW layer-to-layer growth, *W* dimension, and (001) face shows an odd–even effect trend in the *E*_att_ with an overall lowering of the energies as the length increases. The fact that the experimental rates along *W* remain flat (and significantly smaller than along *L*) indicates that, in IPA, the rate-controlling step for the incorporation of the molecules along *W* must be the formation of the HB acid dimer, given the likely solvation of the acid group.

### 2.6 Conformational Free Energy Landscape of DA7C and DA9C in Solution

Taken together, the experimental observations, the attachment energies, and the conformational energy calculations of Figure 1 are consistent with the notion that the *L* dimension ([010]) growth rates are controlled by conformational effects when #C increases above DC7C. To further explore such effects in a more rigorous way and to sample the ability of these diacids to undergo conformational transitions in the liquid phase, we studied the conformational conversions of DA7C and DA9C in IPA using Well-Tempered Metadynamics (WTmetaD) simulations. Full details of these simulations are given in the Methods section.

**Figure 6:**
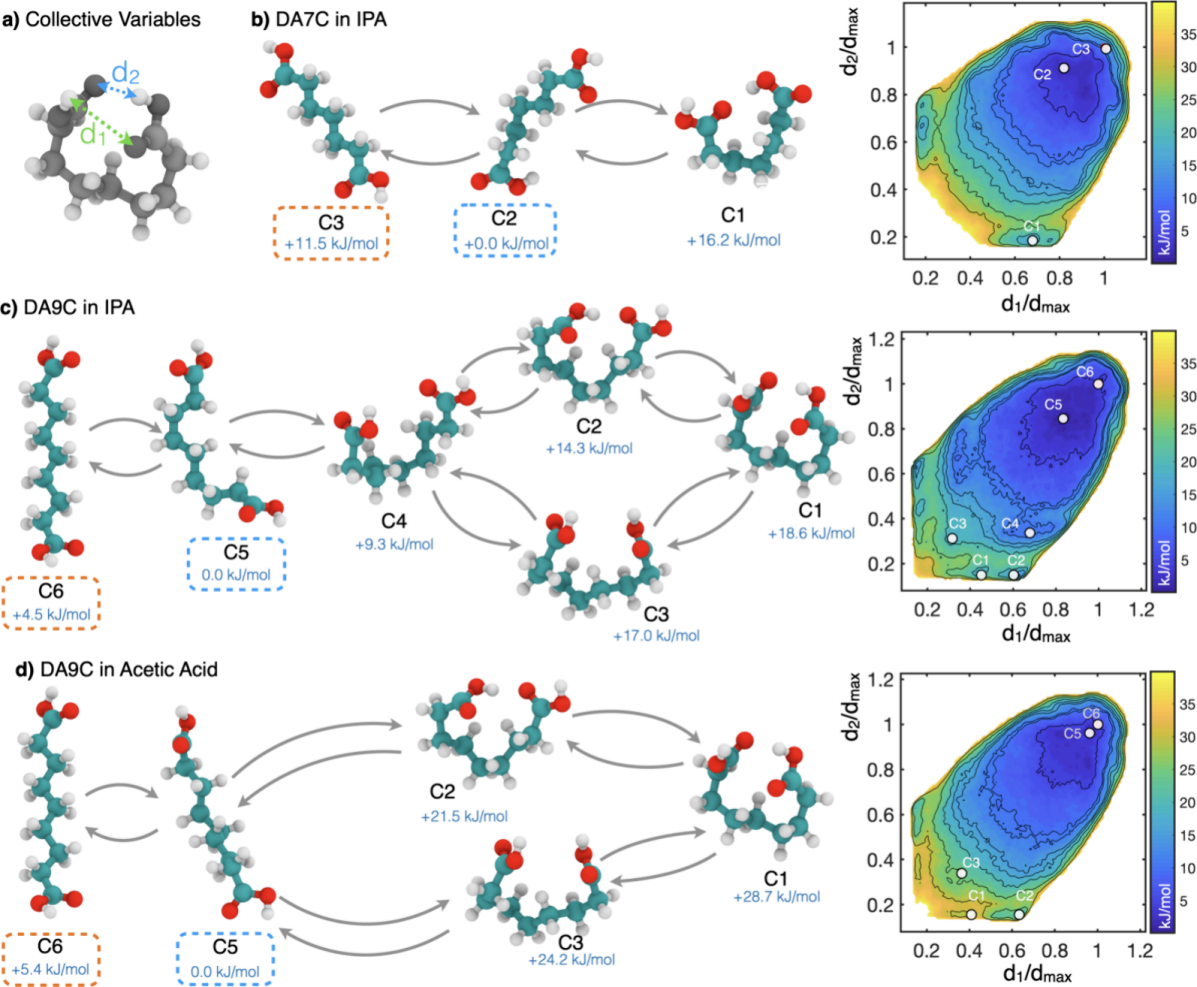
Conformational isomer free energy landscapes of DA7C and DA9C in explicit solutions. (a) Graphical representation of the collective variables (CVs) used to sample and represent the conformational ensembles. (b) Conformational ensemble for DA7C in IPA. (c) Conformational ensemble for DA9C in IPA. (d) Conformational ensemble for DA9C in acetic acid. Representative configurations corresponding to the global minimum of the FES are highlighted in blue. Crystal-like extended conformers in orange.

WtmetaD simulations enable us to obtain a detailed description of the conformational landscape accessible to diacid molecules in explicit solution and estimate the free energy associated with highly populated conformational states. These simulations complement the simpler isolated molecule DFT calculations reported in Figure 1 by inherently accounting for configurational entropy and explicit solvent effects, such as sterical hindrance. Conformational states of both DA7C and DA9C are explored by enhancing the dynamic exploration of two descriptors referred to as collective variables (CVs): *d*_1_ and *d*_2_. The *d*_1_ and *d*_2_ CVs are defined as the distances between the donors and acceptors of the intramolecular H-bonds (see Figure 6a), stabilizing the “closed” diacid conformation (C1 in Figure 6b–d). The free energy surfaces (FESs) obtained sampling *d*_1_ and *d*_2_ are represented as isocontour maps function of the two CVs *d*_1_ and *d*_2_ normalized by *d*_max_, corresponding to *d*_1/2_ in a fully extended crystal-like conformation to facilitate comparisons across different molecular sizes. Low values of *d*_1,2_/*d*_max_ correspond to highly distorted conformations, while *d*_1,2_/*d*_max_ approaching 1 indicates crystal-like configurations. When comparing the conformational FES of DA7C (Figure 6b) and DA9C in IPA (Figure 6c), we note that different systems present key differences.

The first one is related to the conformational distortion of the dominant conformer in solution, which is related to the position of the global minimum of the conformational FES. In the case of DA7C, when compared to the fully extended crystal-like configuration, the global free energy minimum (C2 in Figure 6b) appears minimally distorted. In the case of DA9C, instead, the ensemble of configurations corresponding to the global free energy minimum (Figure 6b, C5) is characterized by significant deviations from the linear crystal-like conformation. This is indicated by a lower distance in CV space between the crystal-like configuration and the global minimum.

To further probe the conformational FES of these systems, we also performed simulations for DA9C (the system with decreased growth kinetics) in acetic acid (Figure 6d), a solvent able to interact strongly with the carboxylic acid groups (making dimers). The IPA and acetic acid simulations are revealing. They show that the dominant conformer in acetic acid is significantly closer to the linear conformer than the dominant conformer in IPA. This indicates that in acetic acid, the probability of finding conformers of DA9C adopting a significantly nonlinear conformation and being prone to give rise to conformational poisoning is significantly lower than in IPA. Moreover, the free energy cost of conformational distortions leading to deviations from a linear conformation is significantly higher in acetic acid than in IPA, where highly distorted, “closed” conformers (C1–4) are associated with free energies up to 10 kJ/mol lower than similar conformations in acetic acid. A second observation relates to the complexity of the conformational ensemble, qualitatively captured by the number of distinct local minima in the FES corresponding to metastable conformational states. While increasing complexity is somewhat expected from longer-chain diacids (i.e., going from DA7C to DA9C), it is noteworthy that the choice of solvent affects the number of DA9C metastable conformers. For instance, in IPA, DA9C displays local free energy minima corresponding to partially folded conformations (indicated with C4 in Figure 6c); however, analogous conformations in acetic acid are not local minima of the FES. This indicates that distorted configurations in IPA are not only more favorable thermodynamically but also likely to be longer-lived compared to similar conformations in acetic acid.

## 3 Discussion

We have measured the experimental growth rates for our series of diacids (DA4C-DA10C) in IPA. Growth along the length of these crystals, dominated by a layer-to-layer stacking of the linear chains, increases steadily with the length of the linear chain of the diacid up to a length of seven carbons. After DA7C, the experimental growth rates drop significantly even though attachment energy calculations indicate that the upward trend in the series should continue as the alkane chain length increases. This drop in rates at DA8C and above must be the consequence of the conformational effects of these diacids in solution.

To prove this hypothesis, we have performed static and dynamic conformational computations. The static calculations (Figure 1) show, as expected, that as the length of the chain is increased, the number of plausible conformers also increases significantly, and folded conformers start to appear for chain lengths above seven carbon atoms. Following these initial computational findings, we explored the conformational landscapes for DA7C and DA9C in explicit IPA using well-tempered metadynamics. In agreement with the static simulations, folded cycled conformers with an intramolecular HB are important in these diacids, with those being significantly more probable at equilibrium in DA9C. Crucially, while DA7C has a fully folded conformer stabilized by an intramolecular HB, it does not have partially folded conformers with no intramolecular HBs (Figure 6). For DA9C, stable folded and partially folded conformers appear in IPA and are accessible by crossing relatively low energy barriers. Further, both sets of simulations agree that as the chain length increases, the global minimum conformer differs more significantly from the crystal conformer. Simulations of DA9C in acetic acid show that the conformational populations are, importantly, dependent on the solvent. In acetic acid, the DA9C linear conformers are more abundant, more similar to the required crystal conformers, and more long-lived than the folded and partially folded conformers.

**Figure 7:**
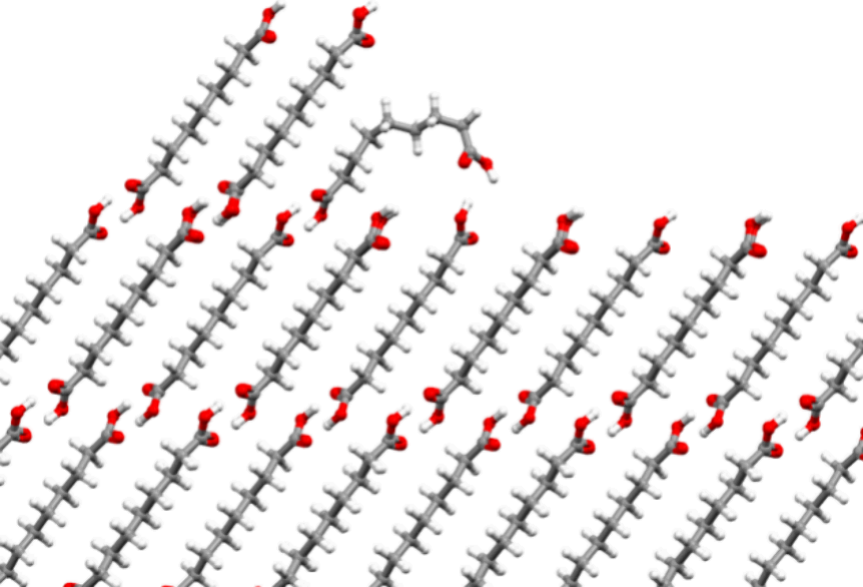
Step on the (010) face of DA9C showing the binding of a “wrong” partially folded conformer. The bulk behind the face is omitted for clarity of viewing. Viewing is along the [010] direction, showing an incomplete step on the (010) surface from the top.

For these “wrong” folded or partially folded conformers to be making such a profound negative impact on the rates along the *L* of the crystal (*b* axis) at DA8C and above, they must be significantly stabilized at growth sites resulting in inhibition of further growth. The exact molecular level steps involved in this process would depend on the growth mechanisms in place for the growth along *L* of these crystals. It seems reasonable to assume stepwise growth, either spiral growth or 2D nucleation, which would be typical at the low supersaturations of 1.25 used in our experiments. We can envisage that when the chain length is long enough (above seven carbon atoms), the “wrong”, *partially folded conformers* might strongly bind to steps present in the (010) face through the formation of two hydrogen-bonded cyclic dimers, as shown in Figure 7. For this mechanism to occur, both surface carboxylic acid groups need to be available for binding, and a partially folded conformer must be present in the solution, which, as we have shown computationally, occurs for DA9C but not for DA7C in IPA. The adsorption and potential “wrong intralayer incorporation” of the *partially folded conformer* would slow the step growth process of the (010) face (the *L* direction) for diacids long enough to be able to access these conformers.

This idea finds tentative support from other complementary work, where measured heats of sublimation (which show no odd–even effect) increase linearly with chain length up to DA8C and after that deviate, decreasing relative to expectations. Ribeiro da Silva et al.^24^ have explained this in terms of folded conformations appearing in the gas phase at these increased chain lengths. In the gas phase, we would expect the fully folded conformers to drive this process, while in solution, the partially folded conformers impact crystal growth. These partially folded conformers indeed exist at levels commensurate with crystal growth inhibition (1% levels) for DA9C but not for DA7C in IPA.

**Figure 8:**
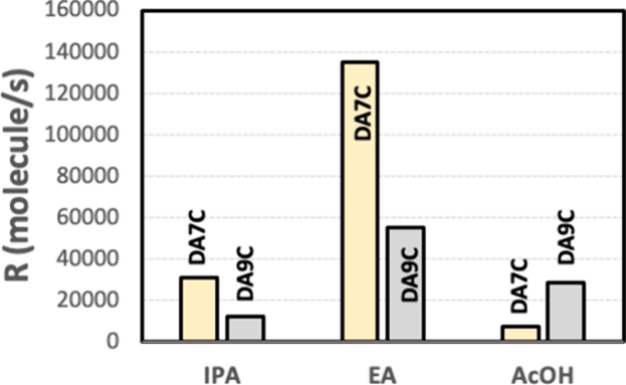
Normalized experimental growth rates for DA7C and DA9C along the length of the crystals in three different solvents: IPA, ethyl acetate (EA), and AcOH.|.

To further prove this mechanism, the learnings from the modeling of DA9C in acetic acid are key. These results show that the partially folded conformers of DA9C are significantly less stable and accessible in acetic acid. Given all of these findings, we returned to the lab to measure the growth rates of DA7C and DA9C from two further solvents: ethyl acetate (EA) and acetic acid (AcOH). EA was chosen for being a solvent with no hydrogen bond donors, and AcOH was chosen because of the simulation results. If the simulation results and our hypothesis of partially folded conformers acting as inhibitors are correct, then the growth of DA9C in acetic acid should not be impacted by conformational self-poisoning. Normalized experimental growth rates for DA7C and DA9C along the *L* dimension of the crystal are given in Figure 8 in the three studied solvents. Rates in EA follow the same trend as in IPA, with DA9C showing a significant drop in the rate relative to DA7C. In AcOH, however, DA9C grows faster than DA7C as it would be expected for a diacid with a longer chain stacking along the length axis or incorporating into steps (Figure 8), with more contributions of van der Waals interactions (Figure 5b).

The growth data for DA7C and DA9C in the various solvents thus prove the validity of our hypothesis and the simulations. DA9C in acetic acid shows a significantly different conformational FES where the partially folded conformation C4 (which has the potential to block the growth of the (010) steps) is no longer a stable conformer of the free energy surface in AcOH, and all other folded conformers become significantly less stable. The simulations shed light on the mechanism of growth inhibition for the long-chain diacids. Partially folded conformers of these diacids, such as C4 found in IPA for DA9C, poison the growth of the diacids along the length by blocking the growth steps through binding with the “wrong” partially folded conformer (Figure 7). To the best of our knowledge, this is the first time that conformational self-poisoning has been demonstrated in the crystal growth of small organic molecules.

## 4 Conclusions

While flexible compounds are known to have a decreased crystallization tendency,^5^ this tendency has often been ascribed to the fact that the “wrong” conformer may be more available in solution than the “right” conformer needed for crystallization. This may indeed be the case for compounds crystallizing with high-energy conformers,^8^ with ritonavir in its form II being such an example.^25^ Indeed, the lack of availability of the “right” conformer in solution may limit its ability to nucleate for the first time, as with ritonavir form II. Here, we have shown a different effect for the first time, whereby the minor populated “wrong” conformer still has a dramatic negative effect on crystallization because it acts as a potent crystal growth inhibitor.

Even though the linear (or nearly linear) conformers of the diacids series are the most stable and most available “right” conformers in solution, the minor “wrong” partially folded conformers (energetically accessible for longer chain acids) play a dramatic negative effect on crystal growth. In fact, even a small amount of the “wrong” conformer can act as a crystal growth inhibitor, thus self-poisoning the crystal growth of the compound. The mechanism of this has been revealed to be the “wrong” minor conformer strongly binding into crystal growth sites, thus dramatically impacting the growth kinetics. Our mechanism and observations align well with a similar effect reported for tautomers,^26^ where minor tautomer concentrations have been found to inhibit the growth of the major tautomer.

Our conformational self-poisoning effect is revealing and daunting since pharmaceutical compounds are becoming ever more flexible and complex^1^ and, as such, becoming more difficult to crystallize. The conformational self-poisoning effect, therefore, is likely to become a ubiquitous problem impacting the crystallization of the drug compounds of the future. For the diacids, we have been able to turn this effect off through a change of solvent from IPA to acetic acid. Solvents that are able to interact more strongly with certain conformers but not others may shift conformational preferences in solution and allow for mitigation of this conformational self-poisoning effect. This strategy needs further exploration for a variety of flexible molecular systems. While conformational self-poisoning in crystal growth is bad news for crystallization scientists, its understanding at the molecular level may lead the way for the development of further strategies to mitigate it.

## Methods

### Experimental Methods

#### Materials and Characterization

All diacids were used as supplied, and deionized water (ASTM D1193-91 Type I) was prepared in the laboratory for immediate use. Isopropanol (IPA, ≥99.5%) and acetic acid (AcOH) were purchased from Sigma-Aldrich Co. Ltd. and used as received.

Single crystals of each diacid were grown by solvent evaporation from several solvents and then used as seeds in crystal growth experiments. It was found that the most suitably sized single crystals for DA4C, DA6C and DA9C were crystallized from IPA, DA5C from acetone, DA7C from deionized water, and DA8C and DA10C from AcOH. Before the growth rate measurements, single crystals were imaged and face indexed for phase and morphological face identification. Face indexing was done on single crystals of 0.5–1 mm in size using a Rigaku Oxford Diffraction FR-X DW diffractometer equipped with Mo Kα X-rays (λ = 0.71073 Å) rotating anode system Varimax microfocus optics. Data were collected in a series of ω-scans at ambient temperature.

#### Growth Rate Measurements

For each diacid (DA4C to DA10C), growth rates were measured in IPA at 20 °C and at a fixed supersaturation ratio *S* (*x*/*x*_sat_) of approximately 1.25. Additionally, growth rates of the DA7C and DA9C diacids were also measured in EA and AcOH at comparable supersaturation values. Prior to each growth experiment, about 30 mL of solution of the desired diacid and solvent and at the desired concentration was prepared in a jacketed vessel and handled carefully to avoid any solvent evaporation. Approximately 1 mL of such solution was then placed in a quartz glass cuvette together with a single crystal seed of the diacid (typically 200 × 500 μm in size), which was then carefully sealed. The cuvette was then placed into a custom-made water bath mounted on an inverted microscope (Olympus CKX41) equipped with a camera, as described previously.^27,28^ The cell was first heated to dissolve the seed crystal slightly, after which the temperature was lowered to 20 °C. After an initial equilibration time, images of the crystal seed were then recorded at elapsed times. The dimensions of both the width and length of seed crystals are plotted as a function of time from which the width and length linear growth rates were derived. At least three independent growth rate measurements were performed for each diacid; these are reported as an average with its standard deviation. Finally, to allow for comparison across the diacids, the derived width and length linear growth rates (in units of length per unit time) were normalized. This was done by dividing the derived rates by each acid’s solubility (in mole fraction) and by the appropriate *d*_hkl_ value (SI) as defined by the crystallography. These two normalizations convert rates from μm/s to molecule/s. The normalized rates then allow for appropriate comparison across molecules with different solubilities and sizes, as discussed elsewhere.^14^

## Computational Methods

### Attachment Energies

Crystal structures of the diacids, taken from the CSD (see Table 1 for refcodes), were fully geometry optimized (allowing the cell to relax) using the COMPASS-II force field^29^ as implemented in the module Forcite in Materials Studio 2019.^30^ Attachment energies were then computed in Materials Studio using the same energy model, normalized per molecule, and then converted to kJ/mol units. Solvent-corrected attachment energies were computed by using a novel procedure under development. Briefly, gas-phase attachment energies were calculated and then multiplied by a correction factor that accounted for the solvent.

**Table 1:** Summary of Conformer Searches, Including Di-acid with Its Number of Rotatable Bonds, Number of Conformers Generated, and Number of Stable Conformers Taken Over for Refinement After Filtering

C#	CSD refcode	rotatable bonds	generated conformers	low energy conformers^a^
4	SUCACB11	5	28	3
5	GLURAC04	6	60	10
6	ADIPAC13	7	113	12
7	PIMELA06	8	312	29
8	SUBRAC05	9	881	28
9	AZELAC05	10	2 207	34^b^
10	SEBAAC07	11	10 000	7^b^

a Conformers within 5 kJ/mol from the most stable conformer.

b The linear crystal conformer was not in this list.

### Sampling of Conformers

Starting from the crystal conformations, conformers of all diacids (DA4C-DA10C) were generated, treating all torsions as flexible and using random sampling techniques as implemented in Materials Studio 2019.^30^ The COMPASS-II force field^29^ with its own charges was used as an energy model throughout the calculations. Sampling was done by disturbing the previously generated conformations across all torsions by a random value between 0 and 180° followed by energy optimization after each generation step. Up to 10,000 conformers per diacid were sampled. The conformational similarity was evaluated by means of RMSD of all atomic positions and making use of symmetry, and duplicates were removed if the RMSD between two conformers was less than 0.375 Å.^8^ Conformers within 5 kJ/mol from the global energy minimum were then taken for refined geometry optimizations. Table 1 summarizes the overall output of the conformational searches.

### Refined Conformational Energies

The lowest energy conformers for each diacid (Table 1) were taken for refined geometry optimization with DFT using the m-GGA TPSS functional with the TNP basis sets and the Grimme van der Waals corrections. A total of 125 conformers were geometry optimized in this step. For DA9C and DA10C, the crystal conformations were not within the lowest energy lists, but they were also taken for DFT optimization. Optimizations were performed using a COSMO implicit solvation model for ethanol (environment most similar to the experimental IPA available computationally). All calculations were done with DMol3 as implemented in Materials Studio 2019.^30^

### Molecular Models for WtmetaD

Molecular systems were modeled in GROMACS^37^ using the General Amber Force Field (GAFF).^31^ Both solute and solvent force field parameters were assigned using the AmberTools suite of programs,^32^ with charges generated at the AM1-bcc level.^36^ The density at 293.15 K (ρ) and enthalpy of evaporation (Δ*H*_vap_) of the pure solvents obtained from unbiased MD simulations, performed following the protocol described in the following section, demonstrated excellent agreement with experimental values (AcOH ρ MD 1073, exp. 1050 kg m^–3^, IPA ρ MD 792, exp. 786 kg m^–3^; AcOH Δ*H*_vap_ MD 51.4, exp. 51.6 kJ mol^–1^; IPA Δ*H*_vap_ MD 44.1, exp. 45 kJ mol^–1^).

### WTmetaD Simulations

The aim of WTmetaD simulations is to estimate the probability of noncrystal conformers as a function of solvent and carbon chain length. As shown in Lucaioli et al.,^17^ the conformational distribution of diacids is weakly dependent on solute concentration. As such, molecular models of isolated diacids solvated in AcOH and IPA were prepared by randomly locating molecules in a cubic simulation box of 8 nm edge length. After the initial energy minimization, performed with the steepest descent algorithm and aimed to avoid unphysical contacts within the simulation box, the temperature and density of the system were equilibrated in the isothermal–isobaric ensemble at 293.15 K and 1 bar for 2 ns. During the equilibration stage, the pressure was controlled with an isotropic Berendsen thermostat. The equilibration stage was followed by a production WTmetaD simulation in the canonical ensemble. In all simulations, the temperature was controlled with the Bussi–Donadio–Parrinello thermostat,^33,34,35^ the real-space cutoff for nonbonded interactions was set to 1.0 nm, and the Particle-Mesh Ewald (PME)^36^ method was used to compute long-range electrostatic interactions. In all simulations, the vibrational motion of hydrogen atoms was constrained by using the LINCS algorithm, enabling an integration time step of 2 fs.

WTmetaD simulations were performed in a two-dimensional collective variable space defined by two distances, *d*_1_ and *d*_2*,*_ corresponding to the distance between the acidic hydrogen in one carboxylic acid moiety and the carbonyl oxygen in the other carboxylic acid moiety. These two collective variables (CVs) were chosen since the conformational preference of dicarboxylic acids is crucially dependent on the formation of intramolecular H-bonds, and the distances *d*_*1*_ and *d*_*2*_ represent the simplest, chemically interpretable low dimensional approximation of the reaction coordinate to form such H-bonded interactions. The two variables are chemically equivalent. However, sampling and projecting the conformational ensemble in (*d*_*1*_*, d*_*2*_) allow the resolution of key representation degeneracies associated with the multitude of arrangements of the two carboxylic groups. A graphical representation of the CVs, together with a representation of the metastable conformational states obtained in CV space, is shown in Figure 6. WTmetaD simulations were performed with a Gaussian width of 0.01 nm for both *d*_*1*_ and *d*_*2*_*,* Gaussian height of *k*_B_*T*, and bias factor of 10. The bias potential was updated every 1000 MD steps. Simulations were extended to 0.5 and 0.25 μs for DA9C and DA7C, respectively, enabling the height of Gaussians to <0.5 kJ/mol. Minimization, MD equilibrations, and WTmetaD simulations were performed in GROMACS^37^ equipped with PLUMED.^38,39^ Convergence of the WTmetaD simulations was monitored by ensuring a reversible exploration of the CV space and producing FESs as a function of simulation time.

## Supporting Information Available

A description of the diacid systems, summary of previous works studying some of the diacids, analysis of the diacid crystal structures, and report of the experimental solubilities and growth rate data including the adopted method for the normalization of rates (PDF)
