# Supplementary material for: Conformational Self-Poisoning in Crystal Growth
Source: JACS Au. 2025 Mar 18;5(4):1781–90. doi: 10.1021/jacsau.5c00043 (PMC12042021; doi:10.1021/jacsau.5c00043)
Supplement: Supplementary file 1 — au5c00043_si_001.pdf [file au5c00043_si_001.pdf]

Supplementary Information

Conformational Self-Poisoning in Crystal Growth

Yumin Liu,[a,b,¶] Veselina Marinova[c,¶], Roger J. Davey,[a] Benjamin Gabriele[a], Matteo Salvalaglio*[c] and Aurora J. Cruz-Cabeza,*[a,d]

[a]Department of Chemical Engineering, University of Manchester, Manchester M13 9PL, United Kingdom.

[b]Beijing National Laboratory for Molecular Sciences, Key Laboratory of Organic Solids, Institute of Chemistry, Chinese Academy of Sciences, Beijing 100190, China.

[c]Thomas Young Centre and Department of Chemical Engineering University College London, London WC1E 7JE, United Kingdom.

[d]Department of Chemistry, Durham University, Durham DH1 3LE, United Kingdom

¶ These authors have contributed equally.

*Corresponding author's email: m.salvalaglio@ucl.ac.uk; aurora.j.cruz-cabeza@durham.ac.uk

1. **Systems** **S2**
2. **Summary of previous work on α, ω-alkanedicarboxylic acids.** **S2**
3. **Structural analysis of structures** **S3**
4. **Measured experimental solubilities in IPA** **S3**
5. **Summary of solubilities** **S6**
6. **Growth rates** **S6**
7. **Normalisation of rates** **S9**
8. **References** **S11**

1. Systems

In this study, we study the growth of the β polymorphs of α,ω-alkane dicarboxylic acids (abbreviated as DA) with total carbon numbers between 4 and 10, in order to explore the impact of increasing chain length and flexibility on the crystal growth rates. The common names for these acids are, in increasing numbers of carbon atoms (#C) from 4 to 10, succinic (DA4C), glutaric (DA5C), adipic (DA6C), pimelic (DA7C), suberic (DA8C), azelaic (DA9C) and sebacic (DA10C) acid. The Cambridge Structural Database (CSD)[1] refcodes used in this work are SUCACB11, GLURAC04, ADIPAC13, PIMELA06, SUBRAC05, AZELAC05, SEBAAC07, respectively. This is an attractive series to choose since growth is characterised by the formation of hydrogen bonds and chain-chain stacking in orthogonal directions. All these di-acids crystallise with Z'=0.5, with the odd ones crystallising in the *C*2/c and the even ones in $P2_1$/c symmetry groups (the stable beta forms). For the odd di-acids, the molecule sits on a screw axis (central carbon atom), and the infinite hydrogen bond chains are constructed through crystallographic inversion and translation. For the even acids, the molecule sits on an inversion centre (central bond), enabling periodic hydrogen bond chains to be constructed through pure translation (Figure 1). For both odd and even acids, chain-chain stacking occurs through translation symmetry along the shortest crystallographic axis (the b-axis). In this context, we wanted to explore whether odd-even effects (observed for their heats of melting[2] solubility[3] and mechanical properties[4]) are also seen in crystal growth and to establish trends for their overall growth kinetics. We first present experimentally measured growth data and its analysis and then present molecular simulations to help interpret such data. We show how increasing the chain length of the acid should lead to an increase in the growth rate, but this trend is eventually broken due to conformational self-poisoning effects.

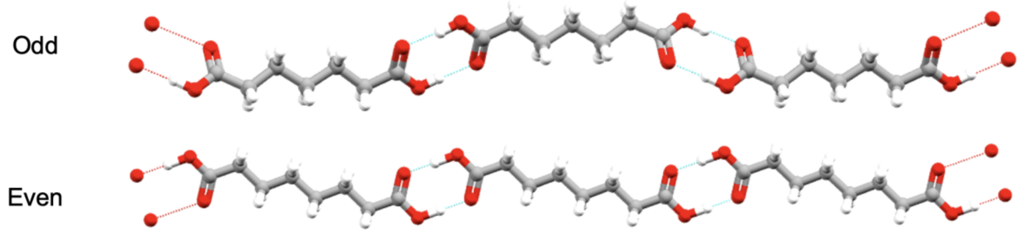

Figure S1. Infinite HB chains as observed in odd (with monomers related by inversion) and even (with monomers related by translation) α,ω-alkanedicarboxylic acids.

2. Summary of previous work on α, ω-alkanedicarboxylic acids.

Of all the molecules in our series (DA4C-DA10C), succinic (DA4C) and adipic (DA6C) acids have been the most studied from the perspective of crystal growth. Davey et al.[5] reported growth rates of the (010) and (001) faces of succinic acid crystals in both water and IPA. Growth rates have magnitudes up to 0.02 μm/s in IPA and up to 0.15 μm/s in water. In the case of adipic acid growing from aqueous solutions, Davey et al.[6] reported rates up to about 0.3 μm/s, suggesting that DA6C grows faster than DA4C. Other than

the growth rates of DA4C and DA6C, significant work has been reported on the impact of monocarboxylic acids on DA6C growth, with reports that additives lead to disorder and twining[7], and DA4C has been subject to morphological prediction.[8]

Studies of the diacids as a series has shown that solubility,[3] mechanical properties[4] and heats of melting[2] of these all show distinct odd-even effects as the chain length increases from 5 to 10 carbon atoms. This trend, in which, for example, odd-numbered chains have lower melting points and are more soluble than the even-numbered ones, is attributed to differences in crystal packing between the odd and the even diacids. According to Thalladi et al.,[2] diacids with an even number of carbon atoms are offset along their length within the columnar H-bonded stacks, whereas such an offset is absent in the diacids with an odd number. Odd members exhibit slightly twisted molecular conformations with severe torsions as opposed to the non-twisted all-*trans* conformations observed in the even members.
While polymorphism is prevalent in these acids, especially in low-molecular-weight ones, this is not considered here since our work focused on the kinetics of crystal growth.[9] All our data refer to the room temperature stable, β forms.

3. Structural analysis of structures

Table S1. Summary of CSD refcodes, space groups and properties for the surfaces at the length, width and thickness growth directions for the series of di-acids (DA4C-DA10C). The angle between the direction of propagation of the hydrogen bond infinite chain, [HB] and the surface under consideration ($\alpha_{(hkl)-[HB]}$) was calculated using Mercury. The surface rugosity (R) and the density of unsatisfied hydrogen bonds per **Å^{-2}** were calculated using the CSD particle module.

			**Length (010)**			**Width (002) or (001)**			**Thickness (100)**		
**#C**	**Refcode**	**SG**	**$\alpha_{(010)-[HB]}$ (°)**	***R* (-)**	**uHB (Å^{-2})**	**$\alpha_{(001)-[HB]}$ (°)**	***R* (-)**	**uHB (Å^{-2})**	**$\alpha_{(100)-[HB]}$ (°)**	***R* (-)**	**uHB (Å^{-2})**
4	SUCACB11	$P2_1/c$	0	1.128	0.000	42	1.473	0.041	46	1.276	0.044
5	GLURAC04	$C2/c$	0	1.468	0.000	40	1.218	0.032	48	1.578	0.041
6	ADIPAC13	$P2_1/c$	0	1.446	0.000	70	2.269	0.054	42	1.445	0.039
7	PIMELA06	$C2/c$	0	1.455	0.000	30	1.215	0.024	62	1.522	0.042
8	SUBRAC05	$P2_1/c$	0	1.406	0.000	60	2.230	0.044	50	1.513	0.039
9	AZELAC05	$C2/c$	0	1.456	0.000	50	1.193	0.019	90	1.509	0.042
10	SEBAAC07	$P2_1/c$	0	1.400	0.000	0	1.119	0.000	47	1.515	0.040

4. Measured experimental solubilities in IPA

Succinic acid (DA4C; Fluorochem, 99%), glutaric acid (DA5C; Alfa Aesar, 99%), adipic acid (DA6C; Sigma Aldrich, 99%), pimelic acid (DA7C; Fluorochem, 98%), suberic acid (DA8C; Alfa Aesar, 99%), azelaic Acid (DA9C; Acros Organics, 98%), sebacic acid (DA10C; Sigma-Aldrich, 99%), acetone (Sigma-Aldrich, ≥99.5%), isopropanol (IPA; Sigma-Aldrich, ≥99.5%), acetic acid (Sigma-Aldrich, ≥99.7%) and

ethyl acetate (Sigma-Aldrich, ≥99.7%) were used as supplied. De-ionized water (ASTM D1193-91 Type I) was prepared in the laboratory for immediate use.

The solubilities of all diacids in IPA at 20 °C were determined gravimetrically. A saturated solution (about 30 mL) with excess desired solute was prepared in a 50 mL double-jacketed glass vessel, which was stoppered and sealed up with parafilm in order to avoid evaporation of solvent during the experimental processes. Solution temperature was kept by a thermostatic bath (Huber Ministat 230, USA) with an uncertainty of ± 0.01 °C. The solution was stirred continuously for roughly 24 h by an electric magnetic stirrer to ensure that the solid-liquid equilibrium was reached. Then the suspension was settled down for another 2 h to get a clear saturated solution before sampling. Afterwards, about 5 mL of the upper clear saturated solution was taken out by a disposable syringe and filtered into a pre-weighed vial quickly to prevent solute from precipitating by a 0.22 μm filter (PES membranes). The vial with saturated solution was weighed by an analytical balance (HM-202, A&D Company, Limited) with a precision of ± 0.0001 g. Finally, the vials were left in the fume cupboard to allow the solvent to evaporate at room temperature and the mass of the vials was recorded periodically until the total weight of the residue did not change. Each experiment was repeated at least five times to check the repeatability and accuracy with the mean values reported. The commercial material, excess samples in solutions and dried products of diacids were tested by PXRD shown in Figure S2. It is indicated that diacids samples were the same crystal form without phase transformation throughout the experiments. Mean values of solubilities with their standard deviations were calculated from multiple repeats (Table S2, Figure S3).

Table S2. Solubilities (in g/kg and mol fraction) of DA4C to DA10C in IPA at 20 °C.

Compound	Solubility (g/kg)	Solubility (mol/mol)	Molarity (mol/L)
DA4C	54.6 ± 0.9	0.02776 ± 0.00045	0.3535
DA5C	557.2± 5.4	0.25340 ± 0.00250	2.5309
DA6C	72.2 ± 0.1	0.02970 ± 0.00002	0.3734
DA7C	334.4 ± 0.9	0.12550 ± 0.00030	1.3686
DA8C	67.4 ± 0.1	0.02326 ± 0.00003	0.2920
DA9C	152.3 ± 0.1	0.04864 ± 0.00002	0.5806
DA10C	52.6 ± 0.1	0.01564 ± 0.00004	0.1979

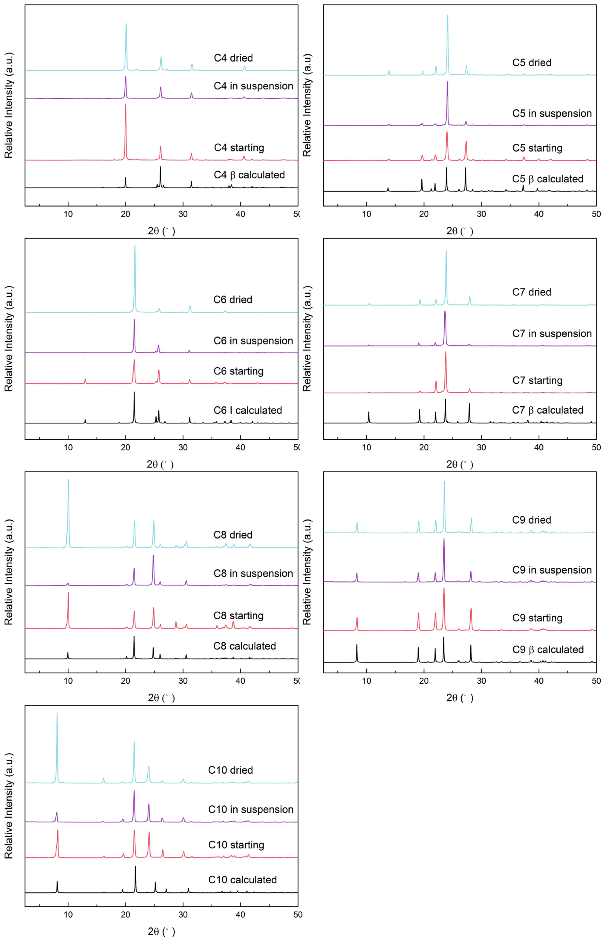

Figure S2 PXRD patterns of diacids of single crystal, starting material, bottom phase and dried product from bottom to top, respectively.

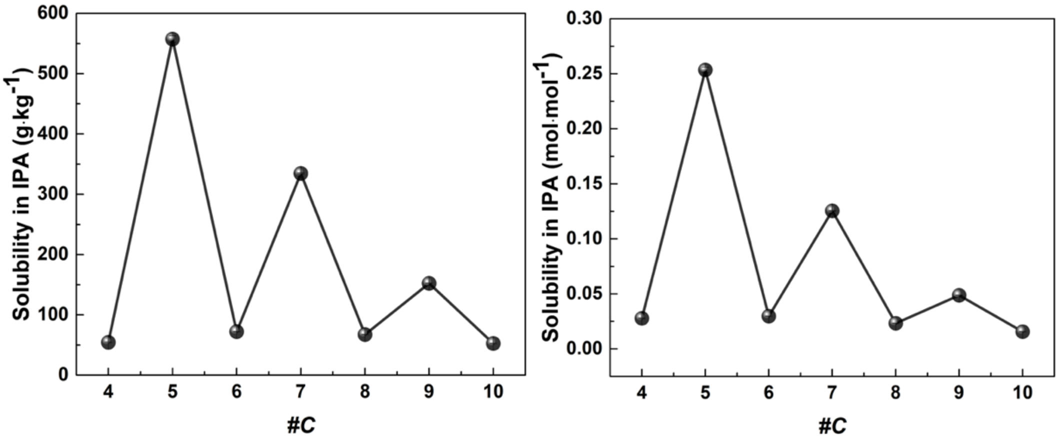

Figure S3 Solubilities (in g/kg left and mol fraction right) of DA4C to DA10C in IPA at 20 °C

5. Summary of solubilities

The solubilities of the dicarboxylic acids in a range of solvents at 20 °C are reported in Figure S4. This figure combines the data in IPA, measured in the current work, with previous literature data[3] in order to show the odd-even trend in solubilities for this series. In the cases of succinic (DA4C) and adipic (DA6C) acids, our data agree well with previous reports 0.029 and 0.027 mol mol^{-1} compared to 0.029 and 0.028 by Davey et al.[5] Overall, the diacids with an odd number of carbon atoms are significantly more soluble than the even-numbered ones, by up to an order of magnitude. In the solvents studied here, the solubility of the diacids goes down as the chain length increases, with the decrease being more significant for the odds than the evens.

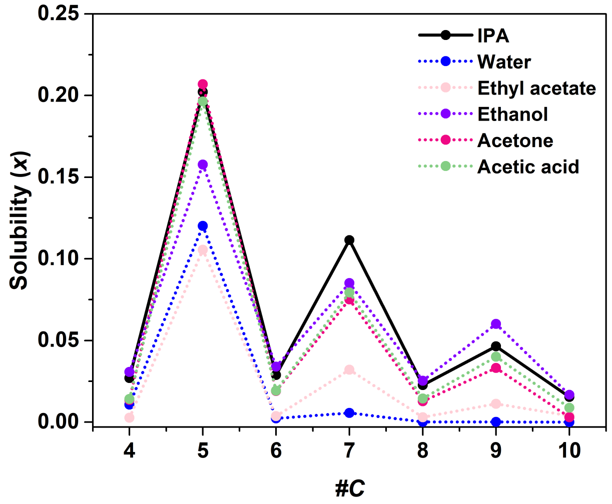

Figure S4. Solubilities (mole fraction) of the di-acids in IPA, water, ethyl acetate, ethanol, acetone and acetic acid at 20 °C.

6. Growth rates

Growth of crystals. Single crystals of all diacids were grown by slow evaporation at room temperature. The slurries for all crystallization experiments were made by DA5C in acetone, DA4C, DA6C and DA9C in IPA, DA7C in de-ionized water, DA8C and DA10C in acetic acid at room temperature, respectively, and leaving them being stirred for about 6 hours. The suspensions were then settled down for approximately 2 h and about 5 mL upper clear solution from each slurry was filtered with a 0.22 μm filter (0.45 μm SC membranes used for acetic acid) into glass vials sealed by parafilms with 2-3 holes punctured in the centre only. All samples were put in the fume hood for several days to allow crystals to appear after the evaporation of solvent.

Face indexing. Indexing crystal faces for single crystals of all diacids (0.5-1 mm in size) were performed using a Rigaku Oxford Diffraction FR-X DW diffractometer equipped with MoKα X-rays (λ = 0.71073 Å) rotating anode system VarimaxTM microfocus optics. The data of space group assignment for the diacids was collected in a series of ω-scans at ambient temperature. Crystal face indices were assigned relative to specific cell setting for each diacids with the aid of a video capture along different directions by CrysAlis Pro software, which is also used for data processing. Miller indices of well-defined faces for diacids were deduced by inspection of the crystal viewed along

specific real and reciprocal space vectors. The preferred orientations for DA8C and DA10C were identified by the PXRD because of the poor quality of crystals. In addition, the face indexing for DA9C was also complemented by the PXRD due to the confusion of polymorphs named in the CSD. The unit cell parameters of single crystals of diacids collected during face indexing are shown in Table S3 (The quality of crystals of DA10C were too poor for face indexing).

Table S3. Unit cell parameters of single crystals of diacids collected during face indexing.

#C		Space Group	*a* / Å	*b* / Å	*c* / Å	*α* / °	*β* / °	*γ* / °
DA4C	SUCACB11	P 2_1/c	5.5261	8.8807	5.1051	90	91.490	90
	Exp		5.0009	8.7735	5.4543	90.244	92.661	89.696
DA5C	GLURAC04	C 2/c	12.968	4.8296	9.982	90	96.872	90
	Exp		12.9660	4.7498	9.6993	89.865	97.657	90.554
DA6C	ADIPAC13	P 2_1/c	7.159	5.1408	9.998	90	110.808	90
	Exp		7.3658	5.1673	10.0475	89.743	110.859	90.177
DA7C	PIMELA06	C 2/c	17.6631	4.7890	9.8882	90	105.612	90
	Exp		9.6210	4.7175	17.6606	89.915	106.081	89.305
DA8C	SUBRAC05	P 2_1/c	8.9800	5.0630	10.1060	90	98.180	90
	Exp		-	-	-	-	-	-
DA9C	AZELAC05	C 2/c	22.5930	4.7804	9.8445	90	109.934	90
	Exp		9.8125	4.7696	21.2968	89.923	95.628	90.033
DA10C	SEBAAC07	P 2_1/c	15.0350	4.9739	10.0660	90	133.290	90
	Exp	-	-	-	-	-	-	-

Growth rate measurement. The growth rate measurements of all diacids in IPA ($S = 1.25$), acetic acid ($S = 1.25$), and ethyl acetate ($S = 1.03$) at 20 °C were carried out by in situ experiments using a growth cell and an inverted microscope (Olympus CKX41) as described previously (Figures S5 and S6).[14-16] The selected supersaturation in experiments was the smallest value, based on that there no primary and secondary nucleation processes happened in the solution. Each experiment here based on a new crystal seed was repeated at least three times to check the repeatability and accuracy. Every seed used for the growth measurement was checked with an assistance of a polar microscope (Zeiss Axioplan 2) in order to make sure that it is a satisfied single crystal to put in the growth cell. All growth rate measurements are on certain crystallographic directions rather than on specific crystal surfaces. The dimensions of both length and width of seed crystal were plotted as a function of time to derive the growth rate from the linear slope (Figures S7 and S8, Tables S4 and S5).

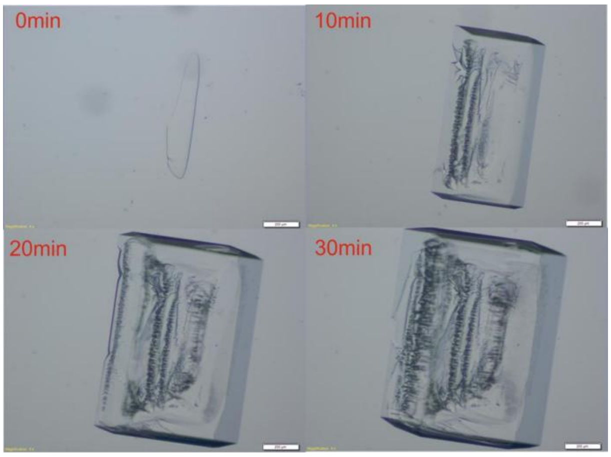

Figure S5. The time lapse sequence of images for seed crystal growing of DA5C at *S* = 1.25 in IPA.

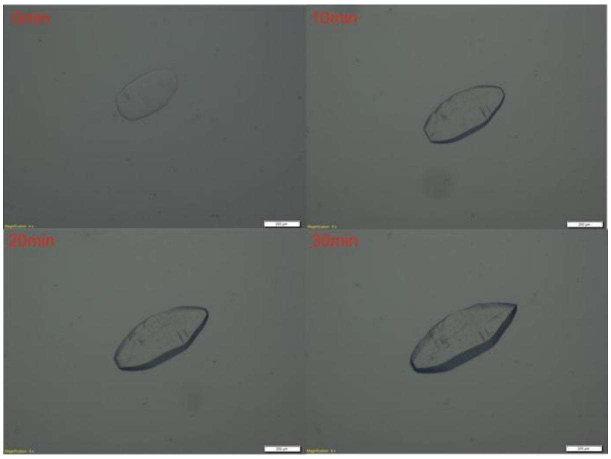

Figure S6. The time lapse sequence of images for seed crystal growing of DA6C at *S* = 1.25 in IPA.

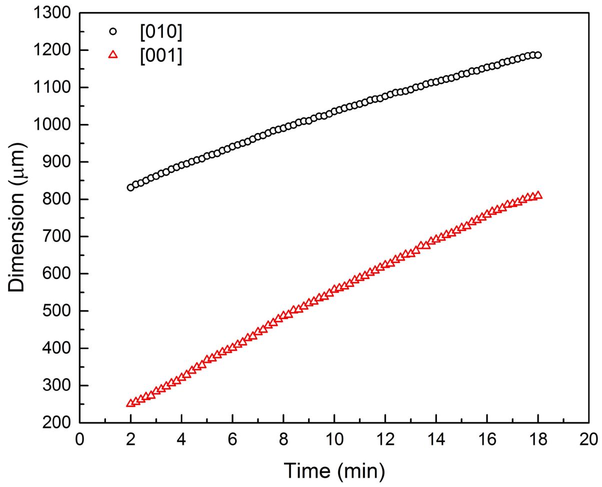

Figure S7. The dimension changes of seed crystal for DA5C as a function of time with supersaturation of 1.25 in IPA at 20 °C.

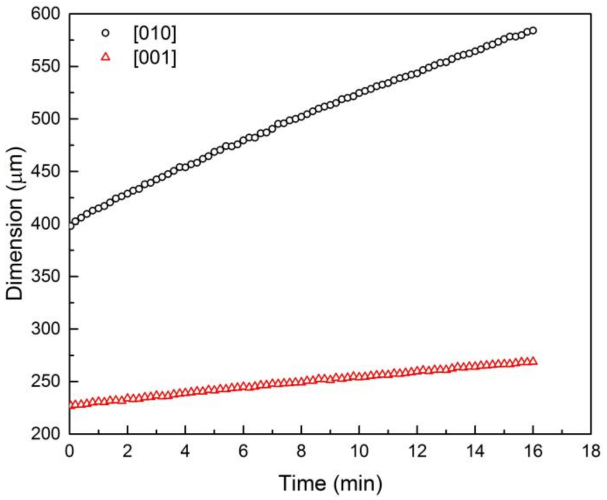

Figure S8. The dimension changes of seed crystal for DA6C as a function of time with supersaturation of 1.25 in IPA at 20 °C.

Table S4. Linear growth rates for diacids DA4C to DA10C in IPA at 20 °C

**Compound**	***R*** **(μm/s)**	***R*** **(μm/s)**
	**[010] - length**	**[001] - width**
DA4C	0.043 ± 0.033	0.077 ± 0.033
DA5C	0.302 ± 0.046	0.489 ± 0.078
DA6C	0.192 ± 0.013	0.045 ± 0.024
DA7C	0.825 ± 0.163	0.139 ± 0.031
DA8C	0.028 ± 0.006	0.025 ± 0.019
DA9C	0.136 ± 0.079	0.123 ± 0.027
DA10C	0.001 ± 0.001	0.048 ± 0.023

Table S5. Linear growth rates for diacids DA7C and DA9C in IPA, ethyl acetate, and acetic acid at 20 °C

**Compound**	**Solvent**	***R*** **(μm/s)**	***R*** **(μm/s)**
		**[010] - length**	**[001] - width**
DA7C	IPA	0.825 ± 0.163	0.139 ± 0.031
DA9C		0.136 ± 0.079	0.123 ± 0.027
DA7C	ethyl acetate	1.033 ± 0.071	0.854 ± 0.084
DA9C		0.437 ± 0.012	0.228 ± 0.017
DA7C	acetic acid	0.134 ± 0.039	0.062 ± 0.008
DA9C		0.269 ± 0.013	0.145 ± 0.032

7. Normalisation of rates

Following Tang et al.,[10] we consider the classic Burton, Cabrera and Frank (BCF) spiral growth rate expression[11] as redefined for solution growth[12,13] in equation 1. The linear growth rate along a specific direction, $G_{[\mathrm{hkl}]}$, is expressed as the product of a solution term, a surface term, and a driving force term. The solution term, is the product of the solubility (x_{sat}) and the rate constant for attachment of molecules at kinks of the

growing surface (k^+). The surface term, is the rate of growth of one step of the (hkl) surface where d_{hkl} is the step height and τ_s^* is the spiral rotation time. The third term is the driving force where σ is the supersaturation ($\sigma = (S - 1)$).

$$G_{[\mathrm{hkl}]} = (k^+ x_{\mathrm{sat}}) \left(\frac{d_{\mathrm{hkl}}}{\tau_s^*}\right) \left(\sigma \ln(1+\sigma)\right) \qquad (1)$$

To enable meaningful comparisons between growth rates of different compounds, one must thus seek to normalise equation 1 in a way so that rates in units of molecules/second can be produced. Accordingly, the linear rates must first be normalised with respect to the varying solubility of each system, x_{sat}, given in table 1. Further, we must acknowledge that, for different molecules and structures, the step height, d_{hkl}, normal to a growth direction will change, so the linear growth rates must also be normalised to account for the number of molecules each step contains. A summary of these normalization values is given in Table S6.

In this way, and with the solubility expressed in mole fraction, we will end up with growth rates having the units *molecule* s^{-1}. Having accounted for these molecular and structural variations enables us to adequately compare growth rates across compounds, allowing for the impact of solvent and molecular structure to be discerned. Of course, it is also true that crystal growth kinetics are controlled not only by molecular structure and related to the interaction energies between molecules on growing surfaces but also by other surface specific factors such as dislocation density, interfacial tension, solvent desorption, etc. However, given that the solvent is fixed and we measure the rates on a reasonable number of different crystals, these effects can be factored out.

Table S6. d_{hkl} for diacids DA4C to DA10C in IPA at 20 °C.

**Compound**	**d_{hlk} (µm/molecule)**	**d_{hlk} (µm/molecule)**
	**[010] - length**	**[001] - width**
DA4C	0.000444	0.000510
DA5C	0.000242	0.000499
DA6C	0.000257	0.000500
DA7C	0.000240	0.000495
DA8C	0.000253	0.000505
DA9C	0.000239	0.000492
DA10C	0.000249	0.000504

8. References

(1) Groom, C. R.; Bruno, I. J.; Lightfoot, M. P.; Ward, S. C. The Cambridge Structural Database. *Acta Crystallogr. Sect. B Struct. Sci. Cryst. Eng. Mater.* **2016**, *72* (2), 171-179. https://doi.org/10.1107/S2052520616003954.

(2) Thalladi, V. R.; Nüsse, M.; Boese, R. The Melting Point Alternation in α,ω-Alkanedicarboxylic Acids. *J. Am. Chem. Soc.* **2000**, *122* (38), 9227-9236. https://doi.org/10.1021/ja0011459.

(3) Zhang, H.; Yin, Q.; Liu, Z.; Gong, J.; Bao, Y.; Zhang, M.; Hao, H.; Hou, B.; Xie, C. An Odd–Even Effect on Solubility of Dicarboxylic Acids in Organic Solvents. *J. Chem. Thermodyn.* **2014**, *77*, 91-97. https://doi.org/10.1016/j.jct.2014.05.009.

(4) Mishra, M. K.; Ramamurty, U.; Desiraju, G. R. Hardness Alternation in α,ω-Alkanedicarboxylic Acids. *Chem. - Asian J.* **2015**, *10* (10), 2176-2181. https://doi.org/10.1002/asia.201500322.

(5) Davey, R. J.; Mullin, J. W.; Whiting, M. J. L. Habit Modification of Succinic Acid Crystals Grown from Different Solvents. *J. Cryst. Growth* **1982**, *58* (2), 304-312. https://doi.org/10.1016/0022-0248(82)90277-9.

(6) Davey, R. J.; Black, S. N.; Logan, D.; Maginn, S. J.; Fairbrother, J. E.; Grant, D. J. W. Structural and Kinetic Features of Crystal Growth Inhibition: Adipic Acid Growing in the Presence of n-Alkanoic Acids. *J. Chem. Soc. Faraday Trans.* **1992**, *88* (23), 3461-3466. https://doi.org/10.1039/FT9928803461.

(7) Williams-Seton, L.; Davey, R. J.; Lieberman, H. F.; Pritchard, R. G. Disorder and Twinning in Molecular Crystals: Impurity-induced Effects in Adipic Acid. *J. Pharm. Sci.* **2000**, *89* (3), 346-354. https://doi.org/10.1002/(SICI)1520-6017(200003)89:3<346::AID-JPS6>3.0.CO;2-I.

(8) Berkovitch-Yellin, Z. Toward an Ab Initio Derivation of Crystal Morphology. *J. Am. Chem. Soc.* **1985**, *107* (26), 8239-8253. https://doi.org/10.1021/ja00312a070.

(9) Lucaioli, P.; Nauha, E.; Gimondi, I.; Price, L. S.; Guo, R.; Iuzzolino, L.; Singh, I.; Salvalaglio, M.; Price, S. L.; Blagden, N. Serendipitous Isolation of a Disappearing Conformational Polymorph of Succinic Acid Challenges Computational Polymorph Prediction. *CrystEngComm* **2018**, *20* (28), 3971-3977.

(10) Tang, S. K.; Black, J. F. B.; Black, S. N.; Cruz-Cabeza, A. J.; Davey, R. J.; Doherty, M. F.; Gabriele, B. P. A. On Comparing Crystal Growth Rates: Para Substituted Carboxylic Acids. *Cryst. Growth Des.* **2023**, *23* (3), 1786-1797. https://doi.org/10.1021/acs.cgd.2c01293.

(11) Burton, W. K.; Cabrera, N.; Frank, F. C.; Mott, N. F. The Growth of Crystals and the Equilibrium Structure of Their Surfaces. *Philos. Trans. R. Soc. Lond. Ser. Math. Phys. Sci.* **1997**, *243* (866), 299-358. https://doi.org/10.1098/rsta.1951.0006.

(12) Snyder, R. C.; Doherty, M. F. Predicting Crystal Growth by Spiral Motion. *Proc. R. Soc. Math. Phys. Eng. Sci.* **2009**, *465* (2104), 1145-171. https://doi.org/10.1098/rspa.2008.0234.

(13) Bhardwaj, R. M.; Price, L. S.; Price, S. L.; Reutzel-Edens, S. M.; Miller, G. J.; Oswald, I. D. H.; Johnston, B. F.; Florence, A. J. Exploring the Experimental and Computed Crystal Energy Landscape of Olanzapine. *Cryst. Growth Des.* **2013**, *13* (4), 1602-1617.

https://doi.org/10.1021/cg301826s.
(14) Liu, Y.; Black, J.; Boon, K. F.; Cruz-Cabeza, A. J.; Davey, R. J.; Dowling, R.; George, N.; Hutchinson, A.; Montis, R. When crystals don't grow-the growth dead zone. *Cryst. Growth Des.* **2019**, *19* (8), 4579-4587. https://doi.org/10.1021/acs.cgd.9b00478.
(15) Black, J. F.B.; Cardew, P. T.; Cruz-Cabeza, A. J.; Davey, R. J.; Gilks, S. E.; Sullivan, R. A. Crystal nucleation and growth in a polymorphic system: Ostwald's rule, p-aminobenzoic acid and nucleation transition states. *CrystEngComm* **2018**, 20, 768-776. https://doi.org/10.1039/C7CE01960B.
(16) Liu, Y.; Gabriele, B.; Davey, R. J.; Cruz-Cabeza, A. J. Concerning Elusive Crystal Forms: The Case of Paracetamol. *J. Am. Chem. Soc.* 2020, *142* (14), 6682-6689. https://doi.org/10.1021/jacs.0c00321.
